# Supplementary material for: Stony coral tissue loss disease induces transcriptional signatures of in situ degradation of dysfunctional Symbiodiniaceae
Source: Nat Commun. 2023 May 22;14:2915. doi: 10.1038/s41467-023-38612-4 (PMC10202950; doi:10.1038/s41467-023-38612-4)
Supplement: Supplementary file 1 — Supplementary Information [file 41467_2023_38612_MOESM1_ESM.pdf]

Stony Coral Tissue Loss Disease Induces Transcriptional Signatures of *in situ* Degradation of Dysfunctional Symbiodiniaceae – Supplementary Information

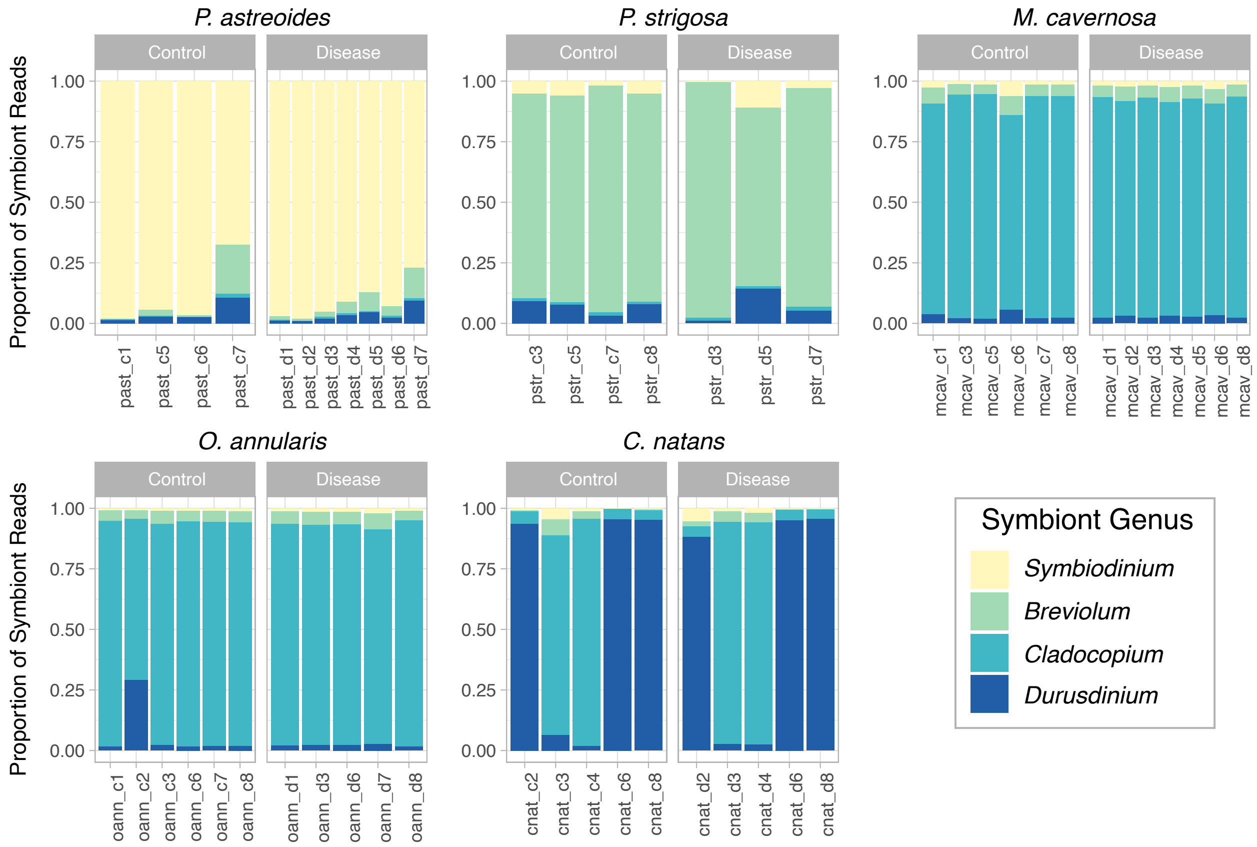

Supplementary Figure 1: Symbiont Composition Within Each Sample Determined by BBSplit. Barplots show the proportion of total symbiont reads that mapped to each *Symbiodiniaceae* reference transcriptome provided. Plots are organized by host species and divided by control and disease (SCTLD exposure) samples. The dominant symbiont in each sample was determined by selecting the genus with the highest proportion of symbiont reads in that sample.

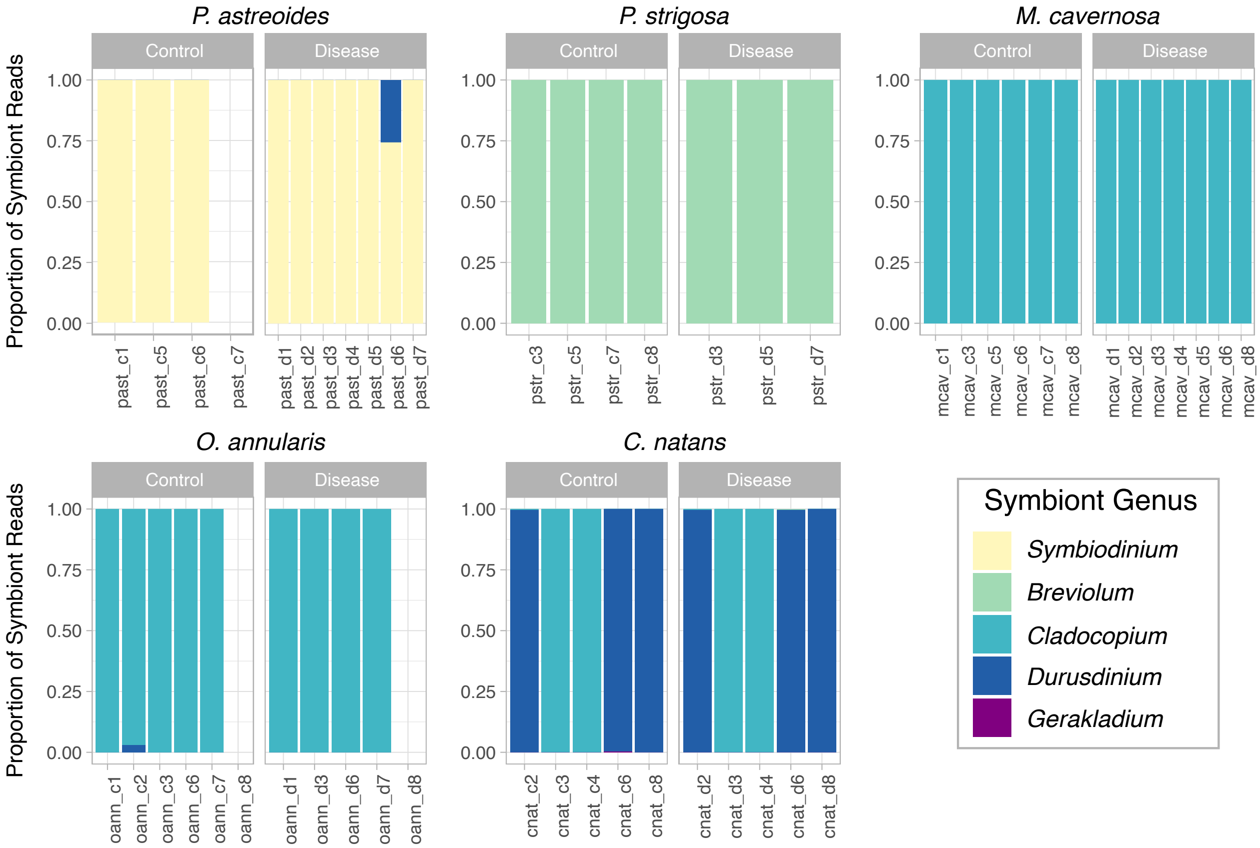

Supplementary Figure 2: Symbiont Composition Within Each Sample Based on Illumina MiSeq of the internal transcribed space-2 (ITS-2) region of Symbiodiniaceae rDNA. Barplots show the relative abundance of hits to a given Symbiodiniaceae genus based on Symportal DIVs. Plots are organized by host species and divided by control and disease (SCTLD exposure) samples.

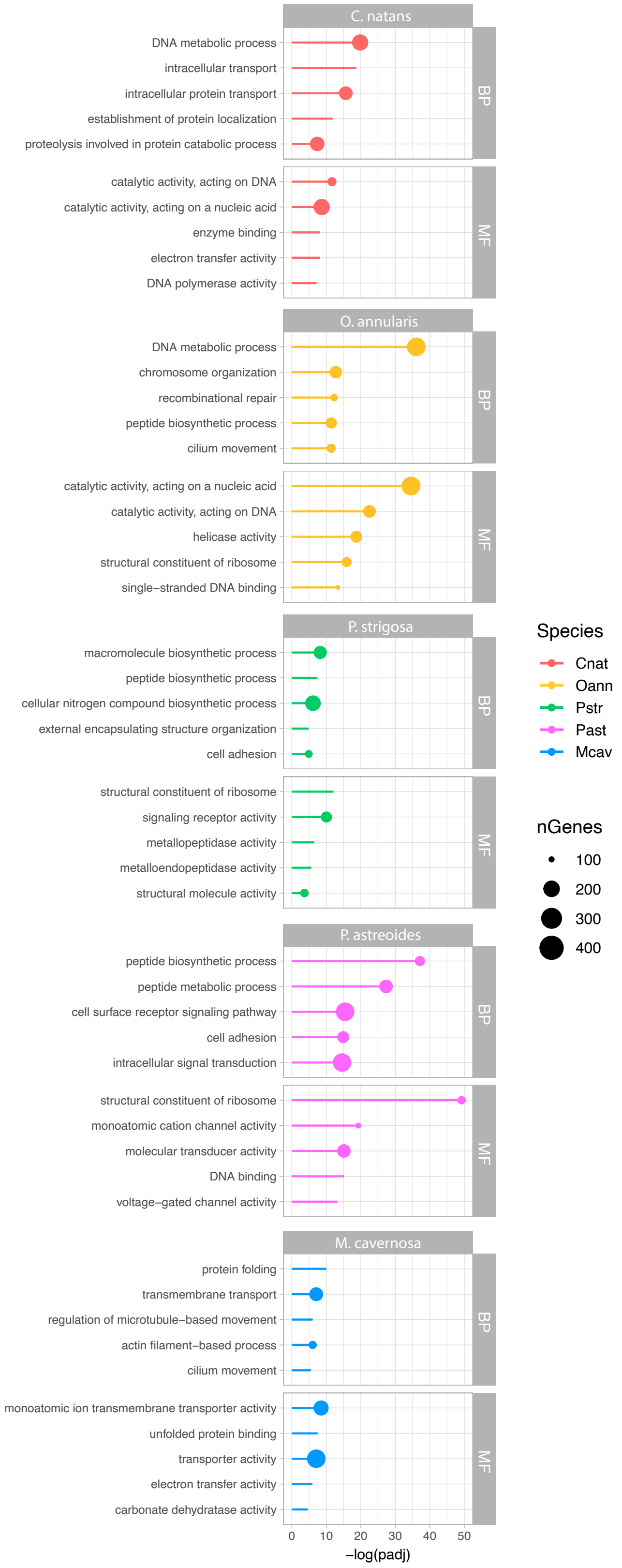

Supplementary Figure 3: GO Enrichment of Coral Species DEGs. The top 5 nonredundant Biological Process (BP) and Molecular Function (MF) GO terms enriched within each species' gene expression using adaptive clustering of GO categories and Mann-Whitney U tests based on log2FoldChange values. Length of the bar represents the -log transformed P-value for the enrichment annotated with that GO term. Size of bubble represents the number of genes within each GO category.

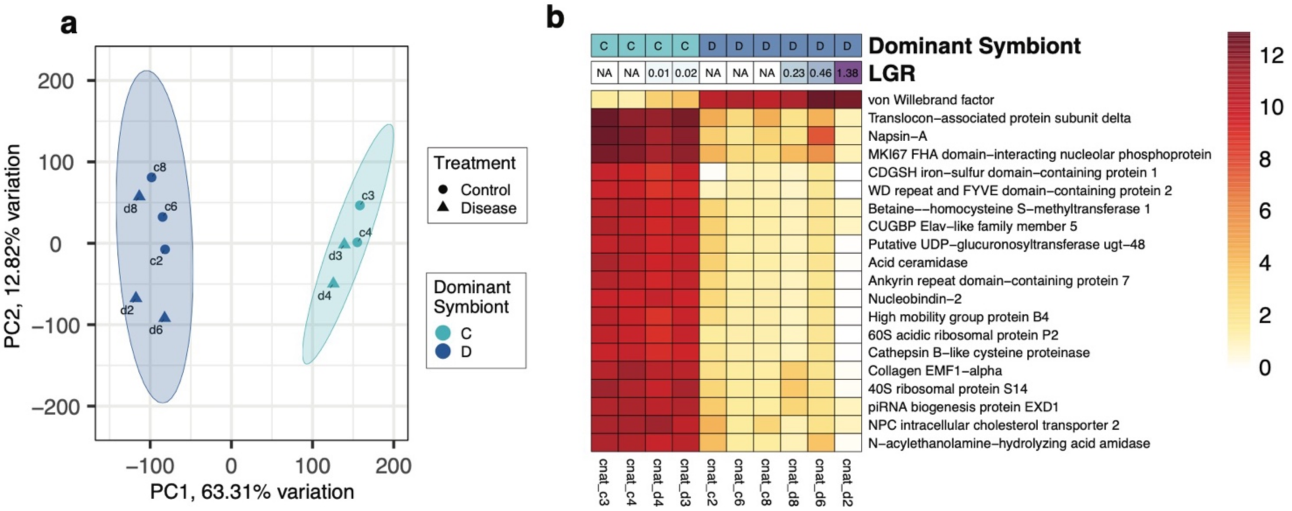

Supplementary Figure 4: Principal Component Analysis (PCA) of *C. natans* expression. **a** PCA of *C. natans* rlog transformed gene expression data from all samples. Note that Principal Component 1 (PC1) explains 63.31% of variance between the two groups of samples and is driven by the dominant symbiont genera within the sample and not by disease treatment. **b** Heatmap plotting the rlog transformed expression of the top 20 PC1 loadings from each sample in (A). These are the *C. natans* genes that are driving the variance within PC1 and have different expression levels based on the dominant symbiont genera within the coral sample. Note that corals hosting *Durusdinium* symbionts had much larger lesion growth rates than corals hosting *Cladocopium*. (C = *Cladocopium*; D = *Durusdinium;* c = control (treatment); d = disease (treatment))

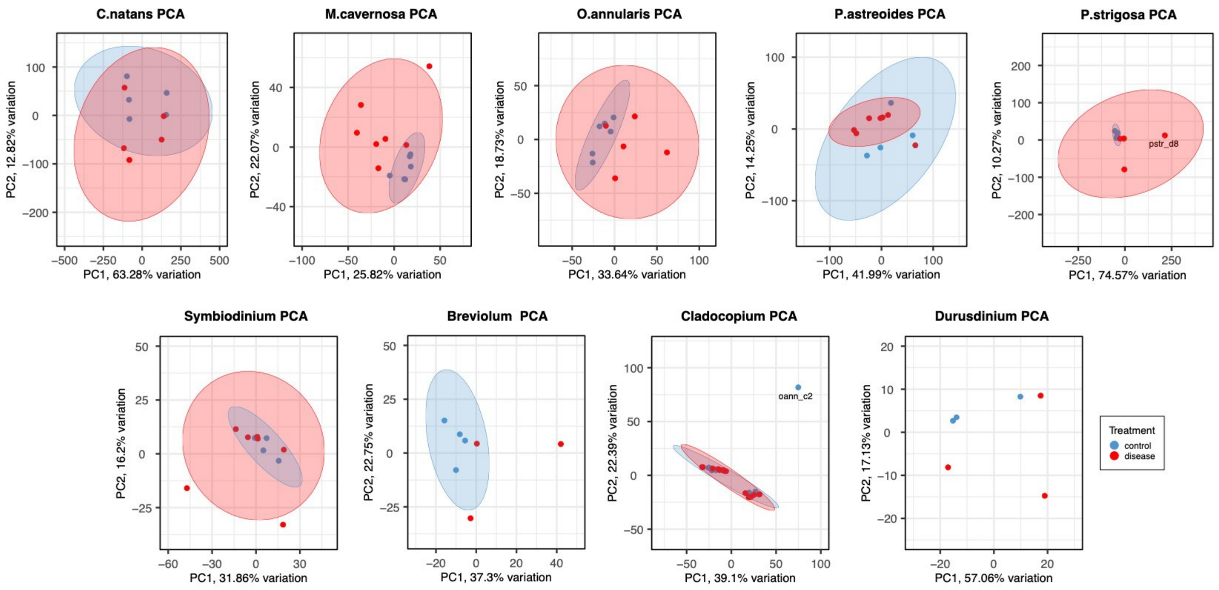

Supplementary Figure 5: Principal Component Analysis (PCA) to detect outliers. PCAs plot the rlog normalized expression of all transcripts with measurable expression within each coral species and dominant symbiont. One sample (pstr_d8) was removed from the coral expression dataset, and one samples (oann_c2) was removed from the symbiont expression dataset.
