# Supplementary material for: Stony coral tissue loss disease induces transcriptional signatures of in situ degradation of dysfunctional Symbiodiniaceae
Source: Nat Commun. 2023 May 22;14:2915. doi: 10.1038/s41467-023-38612-4 (PMC10202950; doi:10.1038/s41467-023-38612-4)
Supplement: Supplementary file 3 — Description of Additional Supplementary Files [file 41467_2023_38612_MOESM3_ESM.pdf]

Description of Additional Supplementary Files

File Name: Supplementary Data 1
Description: Sample metadata and BBSplit mapping results

File Name: Supplementary Data 2
Description: Transmission Experiment results

File Name: Supplementary Data 3
Description: ITS2 sequencing results

File Name: Supplementary Data 4
Description: Transcriptome annotations

File Name: Supplementary Data 5
Description: DESeq2 Results - Coral

File Name: Supplementary Data 6
Description: DESeq2 Results - Symbiont

File Name: Supplementary Data 7
Description: Coral immune homologs – ANOVA results

File Name: Supplementary Data 8
Description: Coral immune homologs – domain structure

File Name: Supplementary Data 9
Description: Coral immune orthologs – ANOVA results

File Name: Supplementary Data 10
Description: Coral EVE results

File Name: Supplementary Data 11
Description: Coral highly variable single-copy orthologs – ANOVA results

File Name: Supplementary Data 12
Description: Symbiont EVE results

File Name: Supplementary Data 13
Description: Symbiont highly variable single-copy orthologs – ANOVA results

File Name: Supplementary Data 14
Description: *Rab7* Correlations
