# Supplementary material for: Stony coral tissue loss disease induces transcriptional signatures of in situ degradation of dysfunctional Symbiodiniaceae
Source: Nat Commun. 2023 May 22;14:2915. doi: 10.1038/s41467-023-38612-4 (PMC10202950; doi:10.1038/s41467-023-38612-4)
Supplement: Supplementary file 5 — Reporting Summary [file 41467_2023_38612_MOESM5_ESM.pdf]

nature portfolio

Corresponding author(s): Laura Mydlarz

Last updated by author(s): Apr 17, 2023

Reporting Summary

Nature Portfolio wishes to improve the reproducibility of the work that we publish. This form provides structure for consistency and transparency in reporting. For further information on Nature Portfolio policies, see our Editorial Policies and the Editorial Policy Checklist.

Statistics

For all statistical analyses, confirm that the following items are present in the figure legend, table legend, main text, or Methods section.

n/a	Confirmed	
☐	☒	The exact sample size (n) for each experimental group/condition, given as a discrete number and unit of measurement
☐	☒	A statement on whether measurements were taken from distinct samples or whether the same sample was measured repeatedly
☐	☒	The statistical test(s) used AND whether they are one- or two-sided<br>*Only common tests should be described solely by name; describe more complex techniques in the Methods section.*
☐	☒	A description of all covariates tested
☐	☒	A description of any assumptions or corrections, such as tests of normality and adjustment for multiple comparisons
☐	☒	A full description of the statistical parameters including central tendency (e.g. means) or other basic estimates (e.g. regression coefficient) AND variation (e.g. standard deviation) or associated estimates of uncertainty (e.g. confidence intervals)
☐	☒	For null hypothesis testing, the test statistic (e.g. F, t, r) with confidence intervals, effect sizes, degrees of freedom and P value noted<br>*Give P values as exact values whenever suitable.*
☒	☐	For Bayesian analysis, information on the choice of priors and Markov chain Monte Carlo settings
☐	☒	For hierarchical and complex designs, identification of the appropriate level for tests and full reporting of outcomes
☐	☒	Estimates of effect sizes (e.g. Cohen's d, Pearson's r), indicating how they were calculated

Our web collection on statistics for biologists contains articles on many of the points above.

Software and code

Policy information about availability of computer code

Data collection

mRNA was sequenced at Novogene (Beijing, China) on the Illumina NovaSeq 6000 for 150 bp, paired-end reads. Raw reads from Novogene were quality filtered and adapter-trimmed using FastP v0.20.1 under default parameters. Genome-guided and de novo transcriptomes were generated, both using Trinity v2.11.0. Coral-only transcripts were obtained from the de novo metatranscriptomes using the in-silico filtration method first outlined by Davies et al. (2016), DOI: https://doi.org/10.3389/fmars.2016.00112. First, the longest isoform was obtained from the metatranscriptomes using the get_longest_isoform_seq_per_trinity.pl script available within the Trinity v2.11.0 package. These isoforms were blasted against a Master Coral database comprised of both genome-derived predicted gene models and transcriptomes spanning a wide diversity of coral families using BlastX v2.2.27. Transcripts with less than 95% identity to this Master Coral database and shorter than 150 bp were filtered out using a custom command-line code (availble in the GitHub repository: https://github.com/kbeavz/SCTLD-Transmission-Experiment-USVI). TransDecoder v5.5.0 was used to first extract the longest open reading frame (ORF) from each transcript and then to generate a predicted peptide sequence from this ORF. Sequences with high sequence similarity within each resulting proteome were then collapsed using cd-hit v4.8.1 under default parameters. These final sequences were extracted from the initial assembly to generate coral-only reference transcriptomes. The completeness of these resulting assemblies was assessedwith Benchmarking Universal Single Copy Orthologs (BUSCO) v5.2.2. BBSplit v38.9.0 was used to separate out coral, Symbiodiniaceae, and non-coral/non-Symbiodiniaceae reads using coral-only and Symbiodiniaceae reference transcriptomes using default parameters. The binning statistics output from BBSplit was used to assess which genera of Symbiodiniaceae was dominant within each sample. Coral and dominant symbiont reads were mapped to their respective transcriptome and quantified using Salmon v1.5.2 with default parameters used for corals and a kmer value of 23 for the dominant symbiont. Host and dominant symbiont transcripts were annotated with reviewed UniprotKB/Swiss-Prot Entry IDs using BlastX v2.2.27 using an evalue cutoff of 10e^-6. Additionally, the predicted proteomes produced after cd-hit were used to identify single copy orthologs across the five coral species and across the four symbiont genera using OrthoFinder v2.5.4 and were annotated with BlastP v2.2.27. Coral host and dominant symbiont transcript abundance was imported into R studio and length-normalized using the R package TXimport v1.16.1.

Data analysis	Transcripts were imported into R Studio v. 2022.02.2 and length-normalized using the package TXimport v1.16.1. The remaining transcripts were regularized log (rlog) transformed and tested for differential expression using the package DESeq2 v1.30.1 with the design "~genotype + treatment" in the host and "~host_species + treatment" in the dominant symbiont. Principal component analysis (PCA) was performed to identify outliers in both the host and symbiont datasets and to illustrate the spatial relationships of gene expression across samples using the R package PCAtools v. 4.2.067. Gene Ontology (GO) enrichment analyses were conducted using adaptive clustering of GO categories and Mann-Whitney U tests based on log2FoldChange values (GO_MWU, https://github.com/z0on/GO_MWU). The function "reduce_overlap" from the R package GOplot v1.0.2 was used to find the top 5 enriched non-redundant Biological Process (BP) and Molecular Function (MF) terms within each species.

For manuscripts utilizing custom algorithms or software that are central to the research but not yet described in published literature, software must be made available to editors and reviewers. We strongly encourage code deposition in a community repository (e.g. GitHub). See the Nature Portfolio guidelines for submitting code & software for further information.

Data

Policy information about availability of data

All manuscripts must include a data availability statement. This statement should provide the following information, where applicable:

- Accession codes, unique identifiers, or web links for publicly available datasets
- A description of any restrictions on data availability
- For clinical datasets or third party data, please ensure that the statement adheres to our policy

Disease phenotype data was collected by Meiling et al. (2021), DOI: https://doi.org/10.3389/fmars.2021.670829.
Raw RNAseq files are available at NCBI SRA PRJNA860922 (https://www.ncbi.nlm.nih.gov/bioproject/PRJNA860922/). Publicly available data used for our analysis include the transcriptomes for Symbiodinium CassKB8 (http://medinalab.org/zoox/), Breviolum minutum (http://zoox.reefgenomics.org/download/), Cladocopium goreaui (http://ssid.reefgenomics.org/download/), and Durusdinium trenchii (https://datadryad.org/stash/dataset/doi:10.5061/dryad.12j173m) as well as the genomes used to generate the M. cavernosa (https://matzlab.weebly.com/data--code.html) and O. annularis (https://www.ncbi.nlm.nih.gov/genome/13173?genome_assembly_id=311351) genome-guided transcriptome assemblies. The Master Coral database used to generate the coral-only de novo reference transcriptomes is publicly available in a Zenodo repository (https://doi.org/10.5281/zenodo.7838980).

Human research participants

Policy information about studies involving human research participants and Sex and Gender in Research.

Reporting on sex and gender	n/a
Population characteristics	n/a
Recruitment	n/a
Ethics oversight	n/a

Note that full information on the approval of the study protocol must also be provided in the manuscript.

Field-specific reporting

Please select the one below that is the best fit for your research. If you are not sure, read the appropriate sections before making your selection.

☐ Life sciences ☐ Behavioural & social sciences ☒ Ecological, evolutionary & environmental sciences

For a reference copy of the document with all sections, see nature.com/documents/nr-reporting-summary-flat.pdf

Ecological, evolutionary & environmental sciences study design

All studies must disclose on these points even when the disclosure is negative.

Study description	Adopting the experimental methodology developed by Williams et al. (2020) and applied by MacKnight et al. (2022), a controlled laboratory disease transmission experiment was carried out in the U.S. Virgin Islands (USVI) and is described fully in Meiling et al. 2021, DOI:https://doi.org/10.3389/fmars.2021.670829. For the control treatment, fragments of six coral species (one fragment each of Colpophyllia natans, Montastraea cavernosa, Orbicella annularis, Porites astreoides, Pseudodiploria strigosa, and Siderastrea siderea) were arranged in a random order equidistant from a healthy colony of Diploria labyrinthiformis. For the disease treatment, corresponding genet fragments of the six experimental species were arranged in a random order around a SCTLD-infected colony of D. labyrinthiformis. This paired design was replicated 8 times (species n = 6, replicates per species n = 8). Corals had no physical contact with other corals. Corals were examined and photographed twice daily (morning and afternoon) to identify the emergence of lesions indicative of SCTLD and assess the visual healthy or corals. When a lesion appeared, the affected coral was monitored at more frequent intervals to determine whether the lesion was actively expanding and appeared consistent with SCTLD. If a lesion was expanding for at least 12 hours, the fragment (as well as its corresponding control genet fragment) was photographed, removed from the mesocosm, and processed for future analyses. The experiment was run for 8 days, at which point, all remaining fragments were processed as above.

Research sample	To obtain healthy coral fragments to be used in a disease transmission experiment, ten visually healthy coral colonies from 5 species of stony coral C. natans, M. cavernosa, O. annularis, P. astreoides, and P. strigosa) were collected from Rupert's Rock reef (a site exhibiting no signs of SCTLD). These five species were chosen based on their known variability in species susceptibility to SCTLD as outlined in the disease case definition (https://nmsfloridakeys.blob.core.windows.net/floridakeys-prod/media/docs/20181002-stony-coral-tissue-loss-disease-case-definition.pdf). Healthy coral samples were then transported to the Center for Marine and Environmental Science (CMES) at the University of the Virgin Islands, fragmented in half with a sterilized chop saw or bandsaw, given identification numbers, photographed, and placed into running seawater tables under shade. These coral fragments were allowed to acclimate for at least 1 week prior to the experiment, and any corals that appeared unhealthy during the acclimation period were not used in the experiment. The samples used in the transmission experiment are meant to represent healthy coral colonies of the above species on a reef prior to SCTLD exposure. Full methods are in Meiling et al. 2021.
Sampling strategy	Research samples, no larger than 25 cm X 25 cm, were collected from Rupert's Rock reef (a site exhibiting no signs of SCTLD) by divers on SCUBA with hammers and chisels. Colonies of the same species were collected > 5 m apart and of different phenotypes when available to maximize intraspecific genetic variation. One day prior to the start of the experiment, ten healthy colonies of D. labyrinthiformis, approximately 20 cm x 20 cm, were collected from Rupert's Rock reef to be used as controls in the experiment. Simultaneously, a separate team of divers collected ten colonies of D. labyrinthiformis, approximately 20 cm x 20 cm,exhibiting visible SCTLD lesions (as according to the NOAA case description) on Flat Cay fringing reef. All samples were extracted using hammers and chisels, placed in individual gallon bags that were sealed, transported to CMES in coolers with seawater where they were then kept separate from other corals until the beginning of the experiment the following day. Diseased corals were observed for 24 hours to confirm the expansion of tissue loss, indicating active disease. All diseased D. labyrinthiformis corals exhibited active lesion expansion and no healthy (control) D. labyrinthiformis corals exhibited lesion development over the duration of the experiment. The sample sizes were selected based on a combination of permit restrictions, ethics, and space in the water tables. These sample sizes allow us to detect differences among species susceptibility.
Data collection	Experimental phenotype data was collected by Sonora Meiling using pencil and paper and imported into Excel (Meiling et al. 2021). The number of fragments with active lesions on each day was used to calculate species-specific disease prevalence. Using the photo of the first observation of a lesion and the last photo of the fragment before processing, lesion progression rate was calculated (cm^2/hour) for each fragment that showed signs of SCTLD. The relative risk of each species was calculated to determine whether there was a significant risk of developing lesions after exposure to SCTLD. The relative risk was identified as the risk in exposed individuals developing signs of SCTLD compared with the risk in non-exposed individuals developing signs of SCTLD throughout the 8-day experiment. Full methods are in Meiling et al. 2021.
Timing and spatial scale	Research sample collection took place on 22 and 26 March 2019. These dates were chosen based on diving conditions. All healthy samples were all collected from the same reef - Rupert's Rock reef, St. Thomas, US Virgin Islands. Colonies of healthy and diseased D. labyrithiformis colonies were collected one day prior to the beginning of the experiment on 3 April 2019 - Flat Cay Fringing Reef, St. Thomas, US Virgin Islands. The experiment began on 4 April 2019 and ended on 12 April 2019. Full methods are in Meiling et al. 2021.
Data exclusions	Some research samples were excluded from our analysis due to the inability to obtain reliable, high-quality RNA (RIN > 4) from those samples. Principal component analysis (PCA) was performed on both coral host and Symbiodiniaceae reads to identify outliers, and one sample within the coral expression dataset (Pstr_d8) and one sample within the Symbiodiniaceae expression dataset (Oann_c2) were identified as outliers and removed. This exclusion criteria was established prior to data analysis.
Reproducibility	Experimental methods are published in detail within Meiling et al. (2021) DOI: https://doi.org/10.3389/fmars.2021.670829. Raw sequencing data is uploaded to NCBI (https://www.ncbi.nlm.nih.gov/bioproject/PRJNA860922) and all code and statistical analyses required to reproduce our results are uploaded into a GitHub repository on Zenodo (https://doi.org/10.5281/zenodo.7839042).
Randomization	Coral fragments were placed into 1 of 8 control mesocosms at random and their corresponding genet fragment was placed into 1 of 8 experimental mesocosms at random. Mesocosms were randomly re-arranged among the three running seawater tables daily. The locations of each research sample within the mesocosms was also randomly rearranged around the central coral each day. A random number generated was used to assign numbered fragments to numbered mesocosms.
Blinding	Blinding was not possible for this experiment. It was necessary to monitor corals so that none of the corals within the control mesocosms developed lesions, as the gene expression of control fragments was directly compared to that of their corresponding genet exposed to SCTLD.

Did the study involve field work? ☒ Yes ☐ No

Field work, collection and transport

Field conditions	All corals used in the experiment were collected from reefs surrounding St. Thomas, U.S. Virgin Islands at depths shallower than 15 m.
Location	Healthy coral colonies were collected from Rupert's Rock Reef (18∘19′39.6′′N 64∘55′33.5′′W) and Diploria labyrinthiformis corals showing signs of SCTLD infection were collected from Flat Cay fringing reef (18∘19′02.9′′N 64∘59′27.0′′W) to be used as disease vectors in the disease transmission experiment. All corals were collected above 15 m depth.
Access & import/export	No more than 10 coral colonies of each species were extracted from each site in order to minimize disturbance to the reefs. Disturbances are also minimized by proper dive training and adhering to collection protocols outlined in the collection permit from the Department of Planning and Natural Resources Coastal Zone Management #DFW19057U (March 4, 2019). This is contribution #229 from the Center for Marine and Environmental Studies at the University of the Virgin Islands.

Disturbance	Minor disturbances can be due to tissue fragmentation and SCUBA operations, but as mentioned above these disturbances were minimized to near zero by proper dive training and adherence to collection protocols.

Reporting for specific materials, systems and methods

We require information from authors about some types of materials, experimental systems and methods used in many studies. Here, indicate whether each material, system or method listed is relevant to your study. If you are not sure if a list item applies to your research, read the appropriate section before selecting a response.

Materials & experimental systems

n/a	Involved in the study	
☒	☐	Antibodies
☒	☐	Eukaryotic cell lines
☒	☐	Palaeontology and archaeology
☐	☒	Animals and other organisms
☒	☐	Clinical data
☒	☐	Dual use research of concern

Methods

n/a	Involved in the study	
☒	☐	ChIP-seq
☒	☐	Flow cytometry
☒	☐	MRI-based neuroimaging

Animals and other research organisms

Policy information about studies involving animals; ARRIVE guidelines recommended for reporting animal research, and Sex and Gender in Research

Laboratory animals	The study did not involve laboratory animals
Wild animals	no wild animals were used in the study
Reporting on sex	Sex information is not relevant to this study.
Field-collected samples	During the transmission experiment, coral fragments were placed into 1 of 8 mesocosms filled with 26 L of filtered seawater, equipped with an air stone, and randomly rearranged among three outdoor shaded seawater tables with chilled running seawater to maintain a constant temperature of ~28C. Seawater at CMES is first pumped from Brewer's Bay up to a sediment settling cistern, then travels by gravity flow to a second sediment settling tank. Next, water is pumped through a filtration system (20 microliter pleated sediment filter) that includes ultraviolet light exposure (80 watt then 40 watt at tables) and then finally out to the running seawater tables. Water in the containers was changed daily (100%). After the experiment, samples were flash frozen and transported to the University of Texas at Arlington where they were stored at -80C until RNA extraction.
Ethics oversight	Department of Planning and Natural Resources Coastal Zone Management

Note that full information on the approval of the study protocol must also be provided in the manuscript.
